# Supplementary material for: The interactome of intact mitochondria by cross-linking mass spectrometry provides evidence for coexisting respiratory supercomplexes
Source: Mol Cell Proteomics. 2017 Dec 8;17(2):216–32. doi: 10.1074/mcp.RA117.000470 (PMC5795388; doi:10.1074/mcp.RA117.000470)
Supplement: Supplemental Data [file supp_RA117.000470_133922_0_supp_23978_fzffwf.zip › spectra_annotation/mito_spectra_annotation/75-1-30-1-9-1.pdf]

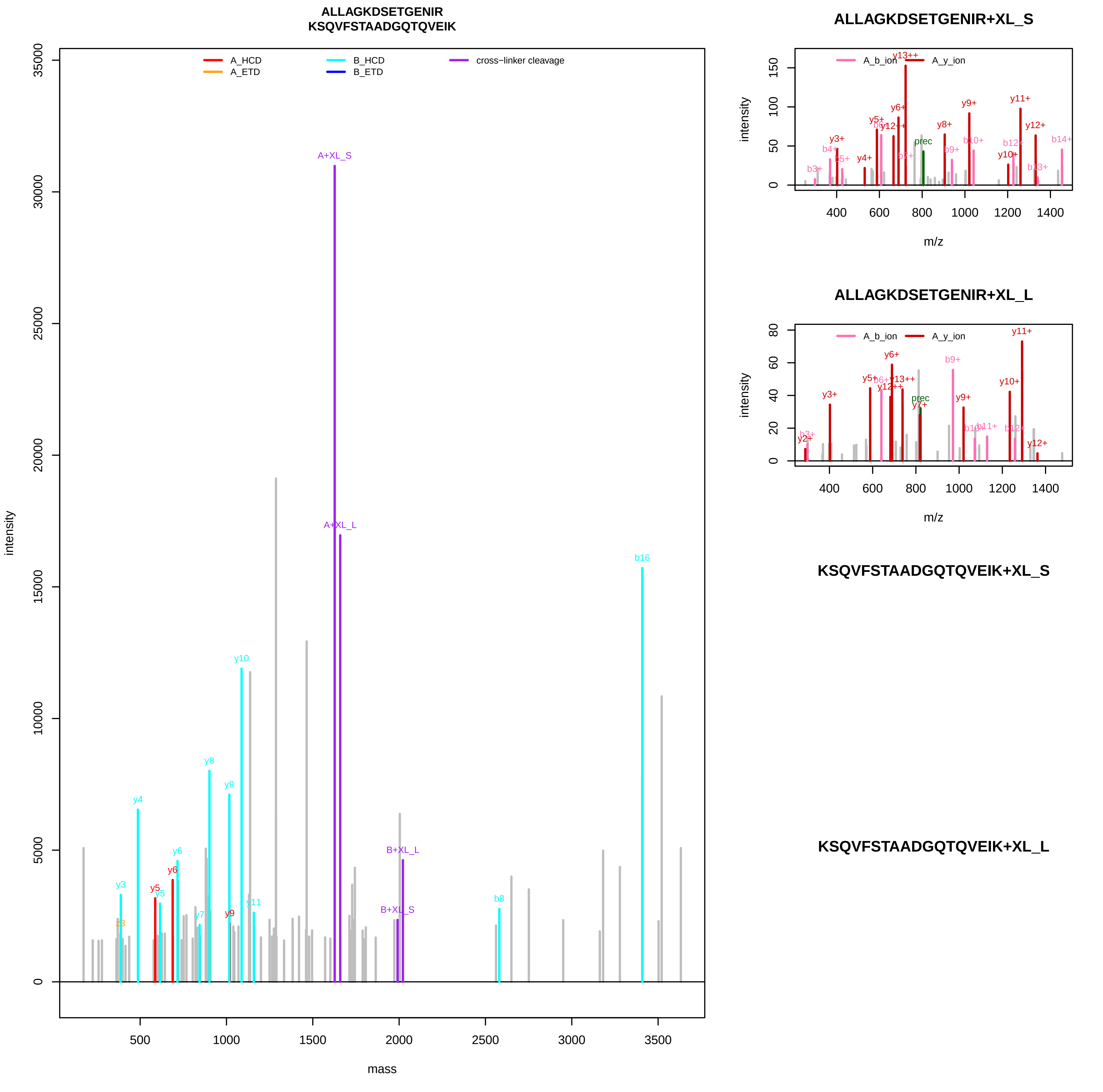

ALLAGKDSETGENIR
KSQVFSTAADGQTQVEIK
A_HCD
A_ETD
B_HCD
B_ETD
cross−linker cleavage
A+XL_S
A+XL_L
B+XL_L
B+XL_S
b16
b8
y3
y4
y5
y6
y7
y8
y9
y10
y11
z3
intensity
mass
0
5000
10000
15000
20000
25000
30000
35000
500
1000
1500
2000
2500
3000
3500
ALLAGKDSETGENIR+XL_S
A_b_ion
A_y_ion
prec
intensity
m/z
ALLAGKDSETGENIR+XL_L
A_b_ion
A_y_ion
prec
intensity
m/z
KSQVFSTAADGQTQVEIK+XL_S
KSQVFSTAADGQTQVEIK+XL_L
